# Supplementary figures and images for: Burkholderia cepacia complex (Bcc) in goats: First report in Bangladesh
Source: PLoS One. 2026 May 22;21(5):e0336003. doi: 10.1371/journal.pone.0336003 (PMC13196930; doi:10.1371/journal.pone.0336003)

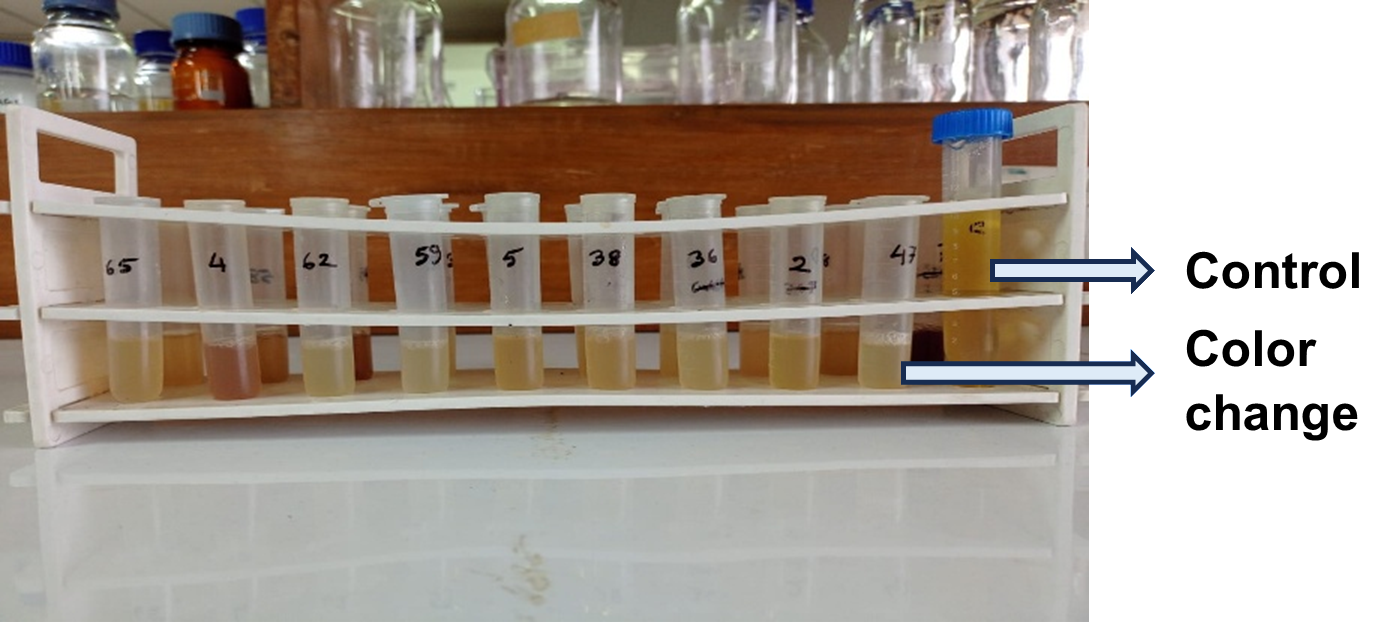

Supplement: S1 Fig — Visible turbidity and distinct color transition in inoculated samples compared to the sterile control. (TIF) [file pone.0336003.s001.tif]

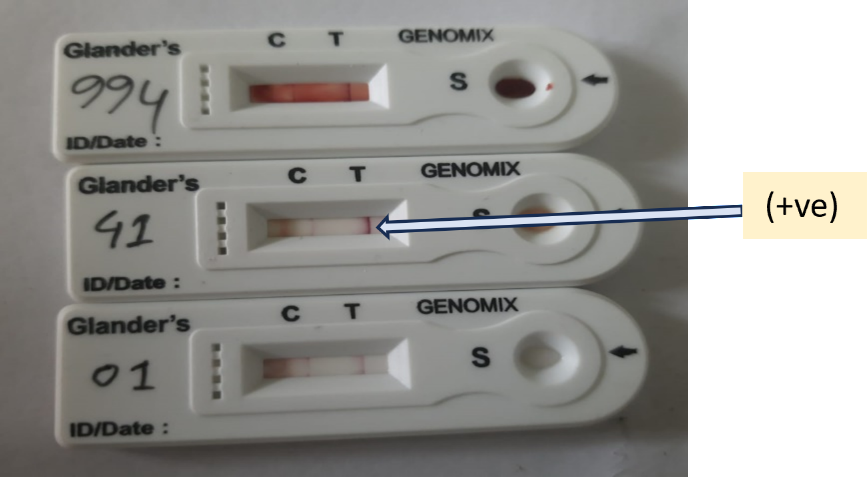

Supplement: S2 Fig — (TIF) [file pone.0336003.s002.tif]

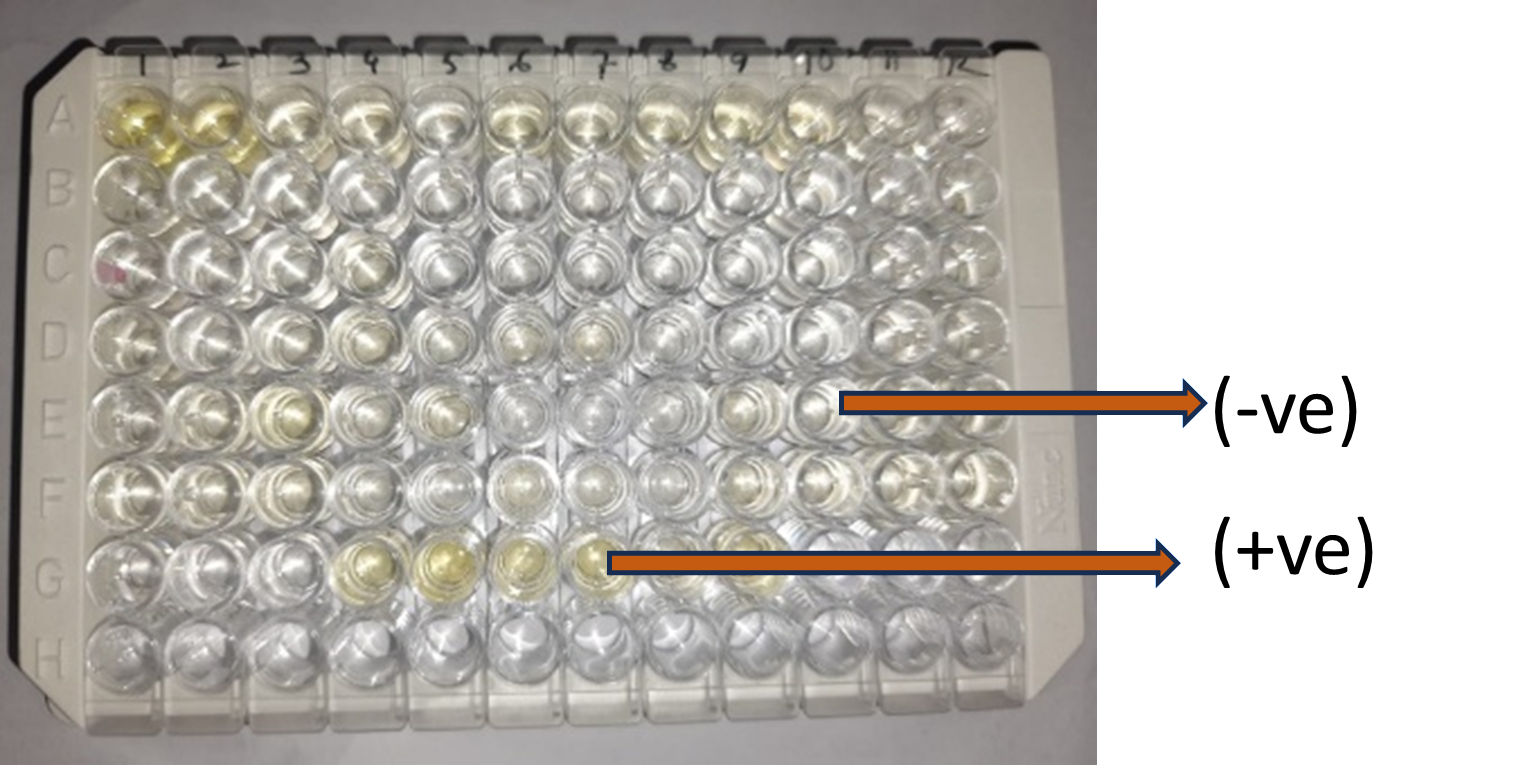

Supplement: S3 Fig — Microplate visualization following the addition of stop solution; yellow coloration indicates positive (+ve) reactivity, while clear wells represent negative (-ve) results. (TIF) [file pone.0336003.s003.tif]
