# Supplementary material for: Burkholderia cepacia complex (Bcc) in goats: First report in Bangladesh
Source: PLoS One. 2026 May 22;21(5):e0336003. doi: 10.1371/journal.pone.0336003 (PMC13196930; doi:10.1371/journal.pone.0336003)
Supplement: Attachment — Submitted filename: BCC Reviewer Response.docx [file pone.0336003.s004.docx]

**Response to the comments of Academic Editor:**

1. **Lack of Abstract**

Reply: Thank you for pointing this out. We have now included a clear and detailed abstract summarizing the study’s objectives, methods, key findings, and significance, highlighting the first detection of *Burkholderia cepacia* complex in goats in Bangladesh and its One Health implications.

2. **Unclear Structure of the Abstract**

Reply: Thank you for the feedback. We have revised the abstract by integrating all sections into a single, cohesive paragraph without separate subheadings to improve clarity and conform to journal guidelines.

3. **Manufacturer Details Missing**

Reply: We acknowledge the editor’s observation concerning the inclusion of manufacturer details for the kits used in the study. In response, we have now added the necessary manufacturer information for all kits utilized in our research. These details are provided in the revised manuscript to ensure full transparency and adherence to the guidelines (line no114-115, 126,157,165,176,192,194).

4. **Vague Terminology for Testing Methods**

Reply: We sincerely appreciate the editor’s remarks related to the vagueness of the term "RAPID TEST KIT." To clarify, we used the Glanders Rapid Detection Test Kit (Genomix Biotech, USA) to validate the presence of B. *cepacia* complex in the original serum samples. This kit was specifically chosen because it detects the *Burkholderia* genus, which includes B. *cepacia*, and is relevant for our study as Glanders is also caused by *Burkholderia* organisms (line no 156-163).

The Glanders Ab Rapid Detection Test was performed according to the manufacturer's instructions. In brief, 10 µL of serum was added to the sample well on the test cassette using the dropper. Two drops of test buffer were placed in the same sample well. After 20 minutes, a line appeared in both the control (C) and test (T) lines, indicating a positive result. A line appearing only in the control (C) region was classified as a negative result.

We have now clarified the specific name and manufacturer of the test kit in the manuscript to ensure greater transparency.

5. **Clarification on PCR Methodology**

Reply: We are thankful for the editor’s constructive comment about for clarification regarding the species-specific PCR. In our study, we performed a multiplex PCR approach to target both the *Burkholderia* genus and B. *cepacia* specifically. The groEL gene PCR assay (139 bp) was used to amplify the *Burkholderia* genus, while the zmpA gene PCR assay (147 bp) was employed to specifically target B. *cepacia* (line no. 185-186, Table-1).

The gel electrophoresis image of the amplicons produced from these assays is presented in the manuscript. In this image, Lane M represents molecular markers, and lanes 1-4 and 5-8 correspond to the samples. Lanes 1-4 are negative, while lanes 5-6 are positive for the *Burkholderia* genus, and lanes 7-8 are positive specifically for B. *cepacia*.

This approach allows for the differentiation between the *Burkholderia* genus and B. *cepacia*, ensuring accurate identification of the pathogen.

6. **Restructure of the Results Section**

Reply: We appreciate the editor’s attention to the issue of the structure of the results section. In response, we have revised and restructured the section to ensure that it is focused on presenting the findings rather than describing the materials and methods. The revised results section now clearly presents the data obtained from the experiments, including the diagnostic tests, PCR assays, and gel electrophoresis analysis, in a more coherent and focused manner.

Thank you for your feedback, which has helped improve the clarity and presentation of our results.

7. **Issues with Data Presentation**

Reply: We appreciate the editor’s comment regarding the alignment of data presentation with scientific conventions. In response, we have carefully reviewed and adjusted the data presentation to ensure it adheres to established scientific standards. This includes proper formatting of tables, figures, and statistical analysis, along with clear and concise descriptions of the results. We have also ensured that the figures and tables are appropriately labeled, with figure legends providing detailed explanations in line with scientific conventions.

8. **Grammatical and Language Issues**

Reply: Thank you for the valuable feedback on the grammatical errors, incorrect spacing, and general language issues in the manuscript. We have thoroughly reviewed and revised the manuscript to correct these errors. The language has been refined for clarity, proper grammar, and consistent formatting. We also addressed issues related to spacing and ensured that the manuscript adheres to the appropriate writing conventions.

Thank you for bringing this to our attention, and we believe these revisions have significantly improved the readability and quality of the manuscript.

9. **Lack of a Control Group**

Reply: We acknowledge the editor’s insightful comment regarding the absence of a control group. In fact, we had included a control group comprising healthy goats from that farm, but this information was unintentionally omitted from the initial manuscript. We have now revised the relevant sections to clearly incorporate and describe the control group and its role in our comparative analysis in line no. 31, 100-101,203-204

10. **Sampling Bias**

Reply: We thank the editor for highlighting the concern about sampling bias due to inclusion of only symptomatic animals. To address this, we had indeed included a control group of healthy goats from the same farms; however, this detail was inadvertently omitted from the original manuscript. We have now revised the manuscript to explicitly describe the control group in line no 31, 100 - 101 and 203- 204 explain how it mitigates potential sampling bias in our study.

In future studies, we will aim to include a broader sample that encompasses both symptomatic and asymptomatic animals to reduce sampling bias and offer a more balanced evaluation of the pathogen’s prevalence and its impact across different animal conditions.

11. **Absence of Antibiotic Susceptibility Testing**

Reply: We sincerely appreciate the editor’s remarks related to the lack of antibiotic susceptibility testing, which is indeed a critical factor in understanding the treatment challenges associated with *Burkholderia cepacia* complex (Bcc). As noted in the manuscript, this study did not include antibiotic susceptibility testing due to financial constraints, which limited the scope of our research. While we acknowledge the importance of testing for Bcc’s resistance profile, the resources available for this study were focused on detecting the presence of the pathogen in the sampled population.

12. **Lack of Environmental Source Analysis**

Reply: We are thankful for the editor’s constructive comment about environmental sources. While we acknowledge that soil, water, and feed are potential reservoirs for Bcc, our study was limited by funding and the inability to obtain samples from farm environments. As B. *cepacia* has been recorded in human clinical cases in Bangladesh, we are concerned about its spread through environmental contamination, potentially affecting livestock. In future studies, we aim to expand the investigation to include environmental sources and secure additional funding and cooperation to address this limitation.

13. **Absence of Statistical Support for Phylogenetic Trees**

Reply: We thank the editor for this important observation. In the revised manuscript, we now clearly indicate that **bootstrap analysis** was conducted to evaluate the robustness of the phylogenetic tree branches. Specifically, we performed **1,000 bootstrap replicates** using the **Neighbor-Joining method** with **Maximum Composite Likelihood distances** in **MEGA11**.

This has been explicitly added to the **Materials and Methods** section, and the corresponding **bootstrap values are now visible at the internal nodes** of the phylogenetic tree (Fig 3). The Results section has also been updated to describe the range of bootstrap values (37% to 100%), highlighting specific relationships supported by higher confidence levels.

Additionally, the **legend for Fig 3** has been revised to clarify that bootstrap support values are displayed at the nodes, and that the sequences generated in this study are marked in red.

14. **No Statistical Analysis**

Reply: We appreciate the editor’s attention to the issue of the lack of statistical analysis. In response to this, we have now included statistical analyses to support and compare the diagnostic findings. The data were analyzed using an Excel 2013 spreadsheet (Microsoft Office 2013, Microsoft, Los Angeles, CA, USA), and binomial 95% confidence intervals were generated using GraphPad Prism version 8.4.3 (GraphPad Software, Inc.) (line no. 196-199). As we have collected samples on just only one farm, no more advance analysis was possible.

**Response to the comment of Reviewer #1:**

**1-** **The study has several methodological limitations that should be addressed to improve its scientific rigor.**

Reply: We appreciate the reviewer’s comment regarding the methodological limitations. The study involved a cross-sectional investigation of 30 goats with the history of abortion and respiratory signs from a farm at Bangladesh Agricultural University (BAU). Blood samples were collected, and bacterial cultures were grown for DNA extraction. PCR assays and phylogenetic analysis were used to identify bacterial species, followed by serological (Glanders Rapid Detection and ELISA) and molecular (PCR with genus-specific and species-specific primers) tests to confirm *B. cepacia*.

**2- Lack of a control group (healthy goats or animals from different farms).**

Reply: We acknowledge the reviewer’s insightful comment regarding the absence of a control group. In fact, we had included a control group comprising healthy goats from that farm, but this information was unintentionally omitted from the initial manuscript. We have now revised the relevant sections to clearly incorporate and describe the control group and its role in our comparative analysis in line no 31, 100 - 101 and 203- 204.

**3- Sampling bias is present, as only symptomatic animals were included.**

Reply: We thank the reviewer for highlighting the concern about sampling bias due to inclusion of only symptomatic animals. To address this, we had indeed included a control group of healthy goats from the same farms; however, this detail was inadvertently omitted from the original manuscript. We have now revised the manuscript to explicitly describe the control group in line no 31, 100 - 101 and 203- 204 and explain how it mitigates potential sampling bias in our study.

In future studies, we will aim to include a broader sample that encompasses both symptomatic and asymptomatic animals to reduce sampling bias and offer a more balanced evaluation of the pathogen’s prevalence and its impact across different animal conditions.

**4- No antibiotic susceptibility testing was performed, despite Bcc’s known resistance profile and clinical relevance, which is critical for understanding treatment challenges.**

Reply: We sincerely appreciate the reviewer’s remarks related to the lack of antibiotic susceptibility testing, which is indeed a critical factor in understanding the treatment challenges associated with *Burkholderia cepacia* complex (Bcc). As noted in the manuscript, this study did not include antibiotic susceptibility testing due to financial constraints, which limited the scope of our research. While we acknowledge the importance of testing for Bcc’s resistance profile, the resources available for this study were focused on detecting the presence of the pathogen in the sampled population.

**5-** **Environmental sources such as soil, water, and feed were not analyzed, leaving potential reservoirs and transmission routes of Bcc uninvestigated.**

Reply: We are thankful for the reviewer’s constructive comment about environmental sources. While we acknowledge that soil, water, and feed are potential reservoirs for Bcc, our study was limited by funding and the inability to obtain samples from farm environments. As B. *cepacia* has been recorded in human clinical cases in Bangladesh, we are concerned about its spread through environmental contamination, potentially affecting livestock. In future studies, we aim to expand the investigation to include environmental sources and secure additional funding and cooperation to address this limitation.

**6- The article does not mention bootstrap analysis, suggesting that the phylogenetic trees were generated without statistical support values.**

Reply: We thank the reviewer for this important observation. In the revised manuscript, we now clearly indicate that **bootstrap analysis** was conducted to evaluate the robustness of the phylogenetic tree branches. Specifically, we performed **1,000 bootstrap replicates** using the **Neighbor-Joining method** with **Maximum Composite Likelihood distances** in **MEGA11**.

This has been explicitly added to the **Materials and Methods** section, and the corresponding **bootstrap values are now visible at the internal nodes** of the phylogenetic tree (Fig 3). The Results section has also been updated to describe the range of bootstrap values (37% to 100%), highlighting specific relationships supported by higher confidence levels.

Additionally, the **legend for Fig 3** has been revised to clarify that bootstrap support values are displayed at the nodes, and that the sequences generated in this study are marked in red.

**7- No statistical analyses were conducted to support or compare diagnostic findings.**

Reply: We appreciate the reviewer’s attention to the issue of the lack of statistical analysis. In response to this, we have now included statistical analyses to support and compare the diagnostic findings. The data were analyzed using an Excel 2013 spreadsheet (Microsoft Office 2013, Microsoft, Los Angeles, CA, USA), and binomial 95% confidence intervals were generated using GraphPad Prism version 8.4.3 (GraphPad Software, Inc.) (line no. 196-199). As we have collected samples on just only one farm, no more advance analysis was possible.

**8- Figure legends lack clarity and descriptive detail, particularly for the gel electrophoresis images, which should be better annotated and presented. Overall, the quality of figures and graphical presentation needs to be improved.**

Reply: We appreciate the reviewer’s feedback on the clarity and descriptive detail of the figure legends, particularly regarding the gel electrophoresis images. To address this, we have revised the figure legends to provide clearer and more detailed descriptions.

- **Fig 2**: Gel electrophoresis image of amplicons produced from the 16S rRNA PCR assay (1400 bp). Lane M represents the molecular markers, and lanes 1-6 correspond to the samples. Lanes 1, 2, 4-6 are positive for the presence of the target bacteria, while lane 3 is negative for unknown bacteria (line no. 208-210).
- **Fig 4**: Gel electrophoresis image of amplicons produced from the groEL gene PCR assay (139 bp) for *Burkholderia* genus and zmpA gene PCR assay (147 bp) for *Burkholderia* *cepacia*. Lane M represents the molecular markers, lanes 1-4 and 5-8 correspond to the samples. Lanes 1-4 are negative, lanes 5-6 are positive for the *Burkholderia* genus, and lanes 7-8 are positive for *Burkholderia cepacia* (line no. 249-253).

In addition, we have enhanced the quality of the figures and improve the overall graphical presentation to meet the standards for clarity and readability.

**Response to the comment of Reviewer #2:**

**1- The manuscript lacks an abstract. The rationale behind providing separate subheadings for objectives, materials & methods, conclusion, and results within what seems to be an abstract is unclear. If these sections are intended as part of the abstract, they should not have separate subheadings. write the answer from my abstract which i have corrected**:

Reply: We value the reviewer’s suggestion on the structure of the abstract. In response, we have revised the manuscript to include a concise, unified abstract. The previous structure, which included separate subheadings for objectives, materials & methods, conclusion, and results, has been adjusted for clarity and coherence. These sections have now been integrated into a single abstract, as is standard for such summaries, to provide a more cohesive overview of the study without the need for distinct subheadings.

Thank you for your constructive feedback, which has helped improve the presentation of our manuscript.

**2- Manufacturer details for any kits used must be provided.**

Reply: We acknowledge the reviewer’s observation concerning the inclusion of manufacturer details for the kits used in the study. In response, we have now added the necessary manufacturer information for all kits utilized in our research. These details are provided in the revised manuscript to ensure full transparency and adherence to the guidelines (line no114-115, 126,157,165,176,192,194). Thank you for your valuable feedback.

**3- The term "RAPID TEST KIT" is too vague. The specific name of the test and its manufacturer should be mentioned.**

Reply: We sincerely appreciate the reviewer’s remarks related to the vagueness of the term "RAPID TEST KIT." To clarify, we used the Glanders Rapid Detection Test Kit (Genomix Biotech, USA) to validate the presence of B. *cepacia* complex in the original serum samples. This kit was specifically chosen because it detects the *Burkholderia* genus, which includes B. *cepacia*, and is relevant for our study as Glanders is also caused by *Burkholderia* organisms (line no 156-163).

The Glanders Ab Rapid Detection Test was performed according to the manufacturer's instructions. In brief, 10 µL of serum was added to the sample well on the test cassette using the dropper. Two drops of test buffer were placed in the same sample well. After 20 minutes, a line appeared in both the control (C) and test (T) lines, indicating a positive result. A line appearing only in the control (C) region was classified as a negative result.

We have now clarified the specific name and manufacturer of the test kit in the manuscript to ensure greater transparency.

**4- For species-specific PCR, clarify whether the primers used amplify the *Burkholderia* agent in general or specifically target B. *cepacia*.**

Reply: We are thankful for the reviewer’s constructive comment about for clarification regarding the species-specific PCR. In our study, we performed a multiplex PCR approach to target both the *Burkholderia* genus and B. *cepacia* specifically. The groEL gene PCR assay (139 bp) was used to amplify the *Burkholderia* genus, while the zmpA gene PCR assay (147 bp) was employed to specifically target B. *cepacia* (line no. 185-186, Table-1).

The gel electrophoresis image of the amplicons produced from these assays is presented in the manuscript. In this image, Lane M represents molecular markers, and lanes 1-4 and 5-8 correspond to the samples. Lanes 1-4 are negative, while lanes 5-6 are positive for the *Burkholderia* genus, and lanes 7-8 are positive specifically for B. *cepacia*.

This approach allows for the differentiation between the *Burkholderia* genus and B. *cepacia*, ensuring accurate identification of the pathogen.

**5- The results section reads more like materials and methods and needs to be restructured accordingly.**

Reply: We appreciate the reviewer’s attention to the issue of the structure of the results section. In response, we have revised and restructured the section to ensure that it is focused on presenting the findings rather than describing the materials and methods. The revised results section now clearly presents the data obtained from the experiments, including the diagnostic tests, PCR assays, and gel electrophoresis analysis, in a more coherent and focused manner.

Thank you for your feedback, which has helped improve the clarity and presentation of our results.

**6- Ensure the data presentation aligns with scientific conventions.**

Reply: We appreciate the reviewer’s comment regarding the alignment of data presentation with scientific conventions. In response, we have carefully reviewed and adjusted the data presentation to ensure it adheres to established scientific standards. This includes proper formatting of tables, figures, and statistical analysis, along with clear and concise descriptions of the results. We have also ensured that the figures and tables are appropriately labeled, with figure legends providing detailed explanations in line with scientific conventions.

Thank you for your feedback, which has guided us in improving the clarity and professionalism of our data presentation.

**7- Figures are not well-represented and lack clarity.**

Reply: We appreciate the reviewer’s feedback regarding the clarity and representation of the figures. In response, we have revised the figures to improve their quality and ensure they are more clearly presented. This includes enhancing the resolution of the images, improving labels, and providing more detailed figure legends for better understanding. We have also ensured that the figures are well-aligned with the data they represent, ensuring greater clarity and easier interpretation.

Thank you for your valuable feedback, which has helped us improve the overall quality of the figures.

**8- The quality of images must be improved to meet publication standards.**

Reply: Thank you. We have improved it.

**9- The manuscript contains grammatical errors, incorrect spacing, and general language issues.**

Reply: Thank you for the reviewer’s valuable feedback on the grammatical errors, incorrect spacing, and general language issues in the manuscript. We have thoroughly reviewed and revised the manuscript to correct these errors. The language has been refined for clarity, proper grammar, and consistent formatting. We also addressed issues related to spacing and ensured that the manuscript adheres to the appropriate writing conventions.

Thank you for bringing this to our attention, and we believe these revisions have significantly improved the readability and quality of the manuscript.

**10- A thorough proofreading and language revision by a native English speaker or a professional editor is recommended.**

Reply: Thank you for your valuable suggestion. In response, the manuscript has been thoroughly revised for grammar, clarity, and academic tone. We are pleased to inform you that the language has been reviewed and refined by **Dr. Garry Adams**, Senior Professor in the Department of Veterinary Pathobiology at **Texas A&M University**, **USA** whose expertise and proficiency in academic English have greatly improved the manuscript's readability and presentation.
