# Supplementary material for: Burkholderia cepacia complex (Bcc) in goats: First report in Bangladesh
Source: PLoS One. 2026 May 22;21(5):e0336003. doi: 10.1371/journal.pone.0336003 (PMC13196930; doi:10.1371/journal.pone.0336003)
Supplement: Attachment — Submitted filename: Response to Reviewers.docx [file pone.0336003.s005.docx]

**Response to Editor Comments**

Dear Dr. Rathore,

Thank you for your detailed and constructive feedback on our manuscript titled *"Burkholderia cepacia complex (Bcc) in Goats: First Report in Bangladesh"* (Manuscript ID: PONE-D-25-28883R1). We appreciate the time and effort you and the reviewers have invested in evaluating our work. Below, we provide our responses to each of the comments raised during the review process:

1. **Please ensure that your manuscript meets PLOS ONE's style requirements, including those for file naming.**

Reply: Thank you for pointing this out. We have reviewed the manuscript formatting and ensured that it now fully complies with PLOS ONE's style requirements, including those for file naming. We have adhered to the specified guidelines and made sure all files are correctly labeled as per the instructions provided.

2. **Thank you for stating the financial disclosure. Please also clarify the role of the funders in the study.**

Reply: We have updated the financial disclosure to reflect the accurate funding source. This research was supported by the University Grants Commission of Bangladesh (UGC) under the project titled "Prevalence and Risk Factors of Glanders in Humans and Horses" (Project Code: 2024/17/UGC) and Ministry of Education (MoE) under the project titled "Development of diagnonis and control strategies against brucellosis in small ruminants and humans" (project code:2022/16/MoE). As per your request, we have added the statement that the funders had no role in the study design, data collection and analysis, decision to publish, or preparation of the manuscript. This updated disclosure has been included in the revised manuscript.

3. **Title Modification: There are several reports on *Burkholderia cepacia* complex (Bcc) in animals, including goats. Therefore, we recommend modifying the title of the paper to reflect this broader context.**

Reply: Thank you for your valuable feedback. As far as I am aware, this is the first report of *Burkholderia cepacia* complex (Bcc) detection in animals, specifically goats, in Bangladesh. While there may be several reports of Bcc in animals worldwide, this study presents the first detection of Bcc in goats within the context of Bangladesh.

Therefore, I would like to retain the focus of the title to reflect this novel finding in Bangladesh. However, based on your suggestion, I have revised the title to better clarify the unique contribution of this study.

Thank you once again for your insightful comments.

4. **Missing Table: Table 1 is not provided. Please ensure that all tables and figures are submitted as separate files as per the submission guidelines.**

Reply: Thank you for your observation. I apologize for the oversight and have now included Table 1 as a separate file (**file name- Table**) under the section other, while also ensuring it is placed in the manuscript after the paragraph in which it is first cited, as per the guidelines.

5. **Figure 2: The marker in Figure 2 is unclear. Kindly provide a clearer gel image with labels indicating all the marker fragments**

Reply: We are thankful for the editor’s constructive comment about for clarification regarding Figure 2. I apologize for the earlier lack of clarity. I have now provided a clearer gel image with labels indicating all the marker fragments, as per your suggestion.

6. **Figure 4: Please label all marker fragments in Figure 4. Additionally, could you clarify why the gel images in Figures 2 and 4 have different backgrounds? Specifically, why does the DNA gel in Figure 4 have a blue background?**

Reply: We appreciate the editor’s attention to the issue of the Figure 4. I have now labeled all the marker fragments in the figure as requested. Additionally, regarding the background color difference between Figures 2 and 4, I have corrected the issue. The gel image in Figure 4 now has a consistent background, and I have ensured that both figures align in terms of presentation.

7. **Control Group Gel Image: Please provide the gel image for the control group used in the detection of Burkholderia cepacia complex (Bcc).**

Reply: Thank you for your comment. I have included the gel image for the control group in Figure 2, where both the suspected animals and the control animals have been tested. I hope this clarifies the inclusion of the control group.

8. **Material and Methods References: Kindly provide the proper citations for all materials, methods, and kits used in this study, including the ladder.**

Reply: Thank you for your valuable feedback. I have now included the proper citations for all materials, methods, and kits used in the study in line no 167, 189, including the ladder in line 192 (https://www.thermofisher.com/order/catalog/product/15628019), as requested. The references have been added to the Materials and Methods section.

9. **PCR Protocol and Bioinformatics Reference: Please include the reference for the PCR protocol as well as the bioinformatics tools used in this study.**

Reply: Thank you for your feedback. I have now included the reference for the PCR protocol, as well as the bioinformatics tools used in this study, in the revised manuscript. These references have been added to the appropriate sections for clarity.

10. **Gene Nomenclature: Please ensure that the writing style of the gene name is consistent and follows the standard conventions**

Reply: Thank you for your valuable feedback. I have now ensured that the writing style of the gene names is consistent throughout the manuscript and follows the standard conventions.

11. **NCBI Submission: Please provide the accession number for the 16S gene data submission to NCBI.**

Reply: Thank you for your comment. The sequences have been deposited in GenBank under the following accession numbers:

- PQ657137.1 (Burkholderia contaminans BAU-MSR-AAM-01)
- PQ657138.1 (B. ambifaria BAU-MSR-AAM-02)
- PQ657139.1 (B. contaminans BAU-MSR-AAM-03)
- PQ657140.1 (B. paraquae BAU-MSR-AAM-04)
- PQ764482.1 (B. cepacia BAU-MSR-AAM-05)

We trust that the revisions made in response to the editor’s and reviewers’ comments have addressed all concerns and enhanced the clarity and quality of the manuscript. We appreciate your thoughtful feedback and look forward to your further guidance.

Kind regards,
Dr. Md. Siddiqur Rahman
Professor, Department of Medicine
Bangladesh Agricultural University, Mymensingh, Bangladesh
Email: siddique.medicine@bau.edu.bd
Contact: +8801918181550
