# Supplementary material for: Burkholderia cepacia complex (Bcc) in goats: First report in Bangladesh
Source: PLoS One. 2026 May 22;21(5):e0336003. doi: 10.1371/journal.pone.0336003 (PMC13196930; doi:10.1371/journal.pone.0336003)
Supplement: Attachment — Submitted filename: Review.docx [file pone.0336003.s006.docx]

Algiers: 31 October 2025

Name of reviewer:

Professor Djamel Baroudi

Review of the manuscript entitled: *'Burkholderia cepacia* complex (Bcc) in goats: First Report in Bangladesh”

The manuscript provides interesting data on the *Burkholderia cepacia* complex bacteria. This is a significant addition to the small number of studies on this neglected pathogen in the existing literature. It makes a significant contribution to the field and offers new interpretations.

2. Manuscript structure:

The methods are sound and clearly described, and are appropriate for the research.

The statistical and results analyses are appropriate and presented accurately and logically.

The conclusions are adequately supported by the results, and the discussion fairly interprets the findings and addresses limitations.

Recommendation: Accept the manuscript for publication.
