# Supplementary material for: Burkholderia cepacia complex (Bcc) in goats: First report in Bangladesh
Source: PLoS One. 2026 May 22;21(5):e0336003. doi: 10.1371/journal.pone.0336003 (PMC13196930; doi:10.1371/journal.pone.0336003)
Supplement: Attachment — Submitted filename: Response_to_Reviewers_auresp_2.docx [file pone.0336003.s007.docx]

**Manuscript ID: PONE-D-25-28883R1**

Title: *Burkholderia cepacia* complex (Bcc) in Goats: First Report in Bangladesh

Journal: PLOS ONE

Dear Academic Editor and Reviewers,

We sincerely thank you for your careful evaluation of our manuscript and for the constructive comments provided. We appreciate the opportunity to revise our manuscript and have addressed all points raised by Reviewer 2 in detail. All revisions have strengthened the clarity, methodological transparency, and scientific rigor of the manuscript.

Below, we provide a point-by-point response to each comment. All changes have been incorporated into the revised manuscript, highlighted in the tracked-changes version, and reflected in the clean version as requested.

**Reviewer 2 Comments and Responses:**

1. **The manuscript requires improvement in English language quality.**

Reply: Thank you for this observation. The entire manuscript has now undergone extensive language revision to improve clarity, grammar, and scientific readability. Sentences have been restructured, redundancies removed, and technical terms clarified. These corrections have strengthened the overall presentation of the manuscript.

2. **Why did the authors use the Rapid Glanders Detection Test for B. cepacia complex? Please clarify.**

Reply: We thank the reviewer for this comment and appreciate the opportunity to clarify. The Rapid Glanders Detection Test Kit was **not used as a confirmatory diagnostic test** in this study. Rather, it was employed as an **initial screening serological assay**. This kit was specifically selected because it detects antibodies against the ***Burkholderia* genus**, which includes *Burkholderia cepacia* complex. Moreover, glanders is also caused by *Burkholderia* species, making this assay relevant for preliminary screening in our investigation.

Importantly, all screening results obtained by this kit were subsequently validated using **ELISA, genus-specific PCR (groEL), species-specific PCR (zmpA), Sanger sequencing, and phylogenetic analysis**, ensuring robust and reliable confirmation of *B. cepacia* complex.

We respectfully note that this clarification was already provided during the **first round of peer review**, and the issue was not raised again in the subsequent review round. In the most recent review, comments were limited mainly to image-related corrections, which were addressed accordingly, and one reviewer accepted the manuscript after revision. As the journal has undergone changes in reviewers across review rounds, we have reiterated and further clarified this explanation here for completeness and to avoid any ambiguity.

3. **Please clarify the criteria used for selecting the animals. Please explain how the authors ensured that other infectious agents were ruled out and describe any clinical or epidemiological criteria used to include animals in the study**

Reply: We thank the reviewer for the comment and appreciate the opportunity to reiterate our response. Animal selection was based on defined **clinical and epidemiological criteria**. The study included goats with a **history of abortion accompanied by respiratory signs**, as these clinical manifestations have been reported in association with *Burkholderia* infections. Clinically healthy goats without any history of respiratory disease or reproductive disorders were included as controls.

All animals originated from a **single, closed herd** with no recent introduction of new animals and were maintained under the same management, feeding, and environmental conditions, ensuring a consistent epidemiological background. Due to logistical and financial constraints, comprehensive screening for all possible infectious agents was not performed. However, no bacterial growth or molecular positivity was observed in the control group, which supports the association of *Burkholderia cepacia* complex with the clinically affected animals. This limitation has been clearly acknowledged in the Discussion section.

We respectfully note that this explanation was already provided during the **first round of peer review** and was not raised again in the subsequent review round. In the most recent review, comments were limited primarily to figure- and image-related corrections, which were addressed accordingly, and one reviewer accepted the manuscript after revision. As the journal has undergone changes in reviewers across review rounds, we have reiterated and clarified this information here for completeness and transparency.

4. **PCR is generally more sensitive than serological assays; however, in this study the PCR positivity rate is lower.**

Reply: We thank the reviewer for this important observation. Although PCR is indeed a highly precise and specific diagnostic tool, a lower prevalence detected by PCR compared to serological assays is biologically and diagnostically expected, particularly in field-based studies.

PCR detects circulating bacterial DNA, which requires the presence of the organism in sufficient quantity at the time of sampling. In contrast, serological assays detect host antibodies, which may persist for extended periods following exposure or during intermittent or low-level infections. Therefore, animals with prior exposure, chronic infection, or transient bacteremia may test positive by serology but negative by PCR.

In the present study, serum was used as the test matrix, and *Burkholderia cepacia* complex is known to cause low-grade or intermittent bacteremia, which can reduce PCR detectability despite true infection. Thus, the lower PCR positivity (25%) compared to serological positivity (75%) reflects the higher specificity of PCR for active circulating infection, rather than reduced accuracy.

This complementary pattern between serological and molecular results is consistent with previous reports and supports the interpretation that PCR provides confirmatory evidence of active infection, while serological assays indicate exposure or immune response. This explanation has been clarified in the revised Discussion section.

5. **Please explain why whole blood was not used for culture and why only serum was selected.**

Reply: We thank the reviewer for this important methodological question. Serum was intentionally selected instead of whole blood to reduce **PCR inhibition and interference from host cellular components**, such as hemoglobin, leukocytes, and intracellular enzymes, which are well known to adversely affect bacterial culture enrichment and downstream molecular detection.

In addition, Burkholderia cepacia complex is an **opportunistic pathogen** that often causes **low-level or intermittent bacteremia**, particularly in chronic or subclinical infections. Serum-based enrichment has been reported to be suitable for detecting such infections, as it minimizes background host DNA and enhances the specificity of molecular assays following culture enrichment.

Given the study design and the need to integrate culture with PCR, Sanger sequencing, and phylogenetic analysis, serum was considered the most appropriate sample matrix.

6. **In the phylogenetic analysis, please specify the outgroup used**

Reply: We thank the reviewer for pointing out this omission. In the phylogenetic analysis, a **non-*Burkholderia* β-proteobacterial 16S rRNA gene sequence** retrieved from GenBank was used as the outgroup to appropriately root the tree and infer evolutionary relationships among the *Burkholderia cepacia* complex isolates. The outgroup was selected based on its phylogenetic proximity (same class) while remaining clearly outside the *Burkholderia* genus, ensuring correct tree polarization and avoiding ingroup bias.

We sincerely thank the Academic Editor and Reviewer for their thoughtful and constructive feedback. We believe that all concerns have been fully addressed and that the revisions have substantially improved the manuscript. We respectfully submit the revised version for reconsideration and hope it now meets the publication criteria of PLOS ONE.

Thank you for your time and consideration.

Yours sincerely,

**Dr. Md. Siddiqur Rahman**

Professor, Department of Medicine
Bangladesh Agricultural University, Mymensingh, Bangladesh
Email: siddique.medicine@bau.edu.bd
Contact: +8801918181550
