# Supplementary material for: Burkholderia cepacia complex (Bcc) in goats: First report in Bangladesh
Source: PLoS One. 2026 May 22;21(5):e0336003. doi: 10.1371/journal.pone.0336003 (PMC13196930; doi:10.1371/journal.pone.0336003)
Supplement: Attachment — Submitted filename: Reviewer Baroudi.docx [file pone.0336003.s008.docx]

For the manuscript intitled "*Burkholderia cepacia* complex (Bcc) in Goats:

First Report in Bangladesh"

The manuscript highlights that the [*Burkholderia cepacia*](https://www.google.com/search?q=Burkholderia+cepacia&newwindow=1&sca_esv=784db34c22f0db79&sxsrf=ANbL-n7ZoaPg2O0dmEWPkAFR6Ixl822Zeg%3A1771452754815&ei=UjmWaYa3Mcusi-gP05KnkQ0&biw=1366&bih=600&ved=2ahUKEwjDz-flh-SSAxVrR_4FHYD6Ac4QgK4QegQIARAB&uact=5&oq=what+is+the+negatif+aspect+in+this+text+bellow%3AThe+Burkholderia+cepacia+complex+%28Bcc%29+comprises+a+diverse+group+of+opportunistic+pathogens%0D%0A25+that+are+critically+relevant+to+human+and+animal+health.+Bcc+induces+life-threatening+infections+of%0D%0A26+the+lung+in+human+patients+with+cystic+fibrosis+and+chronic+granulomatous+disease+and+causes%0D%0A27+mastitis+and+abscesses+in+sheep+and+goats.+Therefore%2C+this+research+aimed+to+identify+the+presence%0D%0A28+of+Burkholderia+cepacia+complex+%28Bcc%29+in+goats+in+Bangladesh+using+serological+tests+such+as+the%0D%0A29+Glanders+Rapid+Detection+Test+Kit+%28GRDTK%29+and+Enzyme-linked+immunosorbent+assay+%28ELISA%29%0D%0A30+and+molecular+tests+such+as+PCR%2C+Sanger+sequencing%2C+and+phylogenetic+analyses.+A+total+of+40%0D%0A31+goat+blood+samples+were+collected%2C+comprising+30+samples+from+goats+exhibiting+a+history+of%0D%0A32+abortion+accompanied+by+respiratory+symptoms+and+10+samples+from+clinically+healthy+control%0D%0A33+goats.+Among+the+30+samples+from+symptomatic+goats%2C+eight+isolates+%2826.67%25%29+were+observed+to%0D%0A34+grow+in+Luria-Bertani+broth%2C+while+there+was+no+growth+from+the+control+group.+Five+%2862.50%25%29%0D%0A35+were+found+positive+for+bacterial+presence+when+analyzed+using+the+16S+rRNA+gene-specific%0D%0A36+PCR+assay+for+broth-positive+samples.+In+the+Genomix+GRDTK+and+ELISA+tests%2C+six+isolates%0D%0A37+were+identified+as+positive+for+Burkholderia+spp.+Of+the+eight+broth+culture-positive+samples%2C+two%0D%0A38+%2825%25%29+were+found+to+be+positive+by+genus-specific+PCR+using+groEL+primers+and+species39+specific+amplification+using+zmpA+primers.+Phylogenetic+analysis+of+positive+goat+samples%0D%0A40+confirmed+the+presence+of+the+Bcc%2C+which+showed+100%25+similarity+with+the+strains+from+India%2C%0D%0A3%0D%0A41+Japan%2C+and) complex (Bcc) is a dangerous opportunistic pathogen causing life-threatening infections in humans and animals, now confirmed for the first time in goats in Bangladesh. It poses a significant threat to animal health (mastitis, abscesses, abortion) and raises critical public health concerns due to its zoonotic potential

- **They are my mains comments:**

**1.** A total of 40 samples (30 symptomatic, 10 controls) is a relatively small sample size, which may limit the statistical power and generalizability of the 26.67% prevalence rate.

**2.**There is a discrepancy between the number of samples positive by different methods: 8 grew in culture, 5 were positive by 16S rRNA PCR, 6 by ELISA/GRDTK, and only 2 by specific *groEL*/*zmpA* PCR. The paper needs to clarify which method is considered the definitive "gold standard" for confirmation.

**3.**While 10 control goats were used, it is not explicitly mentioned if these were from the same farm environment or if they were truly "symptomatic-free" of other respiratory issues that might mimic Bcc.

**4.** The manuscript highlights a significant public health concern, suggesting zoonotic transmission. Deeper information is needed if the study included sampling of the goats' environment (water, soil) or contact humans, or if it only hypothesized the connection.

**5.** The text does not explicitly detail the limitations of the study, such as the cross-sectional design, which cannot determine the direct causality between Bcc and the abortions.

**Thanks**

Pr BAROUDI D.
