# Supplementary material for: Burkholderia cepacia complex (Bcc) in goats: First report in Bangladesh
Source: PLoS One. 2026 May 22;21(5):e0336003. doi: 10.1371/journal.pone.0336003 (PMC13196930; doi:10.1371/journal.pone.0336003)
Supplement: Attachment — Submitted filename: Response_to_Reviewers_auresp_3.docx [file pone.0336003.s009.docx]

**Manuscript ID: PONE-D-25-28883R2**

Title: *Burkholderia cepacia* complex (Bcc) in Goats: First Report in Bangladesh

Journal: PLOS ONE

Dear Academic Editor and Reviewers,

We sincerely thank you for your careful evaluation of our manuscript and for the constructive comments provided. We appreciate the opportunity to revise our manuscript and have addressed all points raised by the Reviewer in detail. All revisions have strengthened the clarity, methodological transparency, and scientific rigor of the manuscript.

Below, we provide a point-by-point response to each comment. All changes have been incorporated into the revised manuscript, highlighted in the tracked-changes version, and reflected in the clean version as requested.

**Reviewer #1 Comments and Responses:**

1. **A total of 40 samples (30 symptomatic, 10 controls) is a relatively small sample size, which may limit the statistical power and generalizability of the 26.67% prevalence rate.**

Response: We appreciate this important observation. We acknowledge that the sample size (n = 40) is relatively small and may limit the generalizability of the findings. However, this study was designed as a preliminary investigation and represents the first report of Bcc in goats in Bangladesh.

We have now clearly addressed this limitation in the Discussion section and emphasized the need for larger, multi-location epidemiological studies to improve statistical power and generalizability

2. **There is a discrepancy between the number of samples positive by different methods: 8 grew in culture, 5 were positive by 16S rRNA PCR, 6 by ELISA/GRDTK, and only 2 by specific groEL/zmpA PCR. The paper needs to clarify which method is considered the definitive "gold standard" for confirmation.**

Response: We thank the reviewer for this valuable comment. The observed differences among diagnostic methods reflect variations in sensitivity, specificity, and biological targets.

We have clarified in the manuscript that bacterial culture followed by molecular confirmation (PCR and sequencing) was considered the gold standard for definitive identification. Additionally, we explained that serological tests (ELISA and GRDTK) indicate exposure rather than active infection.

3. **While 10 control goats were used, it is not explicitly mentioned if these were from the same farm environment or if they were truly "symptomatic-free" of other respiratory issues that might mimic Bcc.**

Response: We appreciate this important comment. We have clarified that all goats were sampled from the same farm environment and that control goats were clinically healthy, showing no signs of respiratory or systemic illness.

4. **The manuscript highlights a significant public health concern, suggesting zoonotic transmission. Deeper information is needed if the study included sampling of the goats' environment (water, soil) or contact humans, or if it only hypothesized the connection**

Response: We thank the reviewer for this important and insightful comment. We would like to clarify that environmental (soil, water) and human sampling were not included in the present study, and therefore the potential for zoonotic transmission was hypothesized based on existing literature and the known ecological and pathogenic characteristics of the Burkholderia cepacia complex (Bcc).

Relevant evidence regarding environmental reservoirs and human infections of Bcc has been discussed in the Introduction section with appropriate references. To avoid overinterpretation, we have now revised the manuscript to clearly state that zoonotic transmission was not directly investigated in this study and remains hypothetical.

5. **The text does not explicitly detail the limitations of the study, such as the cross-sectional design, which cannot determine the direct causality between Bcc and the abortions.**

Response: We appreciate this important comment. We have now expanded the limitations section to include the cross-sectional design, which does not allow causal inference, as well as other limitations such as small sample size, single-farm sampling, lack of environmental/human sampling, and absence of antimicrobial susceptibility testing. These additions have been incorporated into the Discussion section.

We sincerely thank the Academic Editor and Reviewer for their thoughtful and constructive feedback. We believe that all reviewer concerns have been fully addressed and that the revisions have substantially improved the manuscript. We respectfully submit the revised version for reconsideration and hope it now meets the publication criteria of PLOS ONE.

Thank you for your time and consideration.

Yours sincerely,

**Dr. Md. Siddiqur Rahman**

Professor, Department of Medicine
Bangladesh Agricultural University, Mymensingh, Bangladesh
Email: siddique.medicine@bau.edu.bd
Contact: +8801918181550
